# Reviewing COVID-19 Modelling amidst Recent United States Protests

**DOI:** 10.5334/aogh.2970

**Published:** 2020-07-06

**Authors:** Luther-King O. Fasehun

**Affiliations:** 1 Temple University College of Public Health, Philadelphia, PA, US

## Abstract

The unfortunate death of George Floyd in Minnesota, following police brutality, is deeply regrettable, and the ensuing protests in cities across the United States bring up issues on the potential impacts of the protests on the epidemiology of COVID-19 in the United States. Modelling scientists will need the best time-series estimates of the numbers of protesters in every city where protests took place; the length of time the protests were active, and what distance and routes were covered by the protesters; and the numbers and distribution of security personnel deployed to keep the protests safe, as well as curtail the chaotic exacerbations that were reported across many areas.

To the Editor:

The unfortunate death of George Floyd in Minnesota, following police brutality, is deeply regrettable, and the ensuing protests in cities across the United States bring up a wide range of issues. This letter will focus on the potential impacts of the protests on the epidemiology of COVID-19 in the United States. Its goal is to stimulate research based on methodologically sound approaches for COVID-19 modelling to assess the effects of the protests on spread of disease.

As of 31 May, 2020, the United States had recorded 1,786,593 cases and 104,319 deaths from COVID-19 [1]. The states with the most cases, in decreasing order, are New York, New Jersey, Illinois, California, and Massachusetts [2]. It is worth noting that while New York, New Jersey, and Illinois were starting to report declining cases, the past two days have started to show an uptick [3]. These are states with metropolitan areas that have witnessed protests, photos of which have shown weak social distancing measures. To this end, an urgent re-consideration of the modelling estimates of COVID-19 in the US are necessary.

Mathematical modelling of infectious diseases has been implemented by different experts in different disease scenarios, from Ebola to HIV to COVID-19. The conceptual underpinnings of effective infectious disease modelling aim to describe the non-linear *transmission process* [4] as close to reality as possible, given prevailing circumstances. For COVID-19, specifically, epidemiologists and other scientists have developed models in several waves, from the initial outbreak in China, to global spread in Europe and the United States, to the effects of gradual easing of lockdowns around parts of the world on the transmission of the disease. In a recent PubMed (National Center for Biotechnology Information, Bethesda, MD) search using the keywords “COVID-19,” “model,” and “United States” in article titles, a total of 13 articles were retrieved. In reading through the abstract and methods section of all articles, it was found that none considered the rising number of Americans returning to the homeland. As of 13 May, 2020, the United States Department of State reported that it had repatriated 85,141 Americans from 131 countries and territories, including China. In light of the recent protests, modelling scientists will need the best time-series estimates of the numbers of protesters in every city where protests took place; the length of time the protests were active and what distance and routes were covered by the protesters; and the numbers and distribution of security personnel deployed to keep the protests safe as well as curtail the chaotic exacerbations that were reported across many areas.

Scientists can also glean from previous modelling efforts that have been performed in the contexts of chaotic situations like the Ebola Virus Disease (EVD) happening within the setting of armed conflict in parts of the Democratic Republic of Congo (DRC) [5]. While it is hoped that the protests will wane soon, it is crucial that COVID-19 modelling estimates accurately account for this important dynamic as much as possible.
